# Disk Battery as a Vaginal Foreign Body in a Five-Year-Old Preadolescent Child

**DOI:** 10.7759/cureus.13727

**Published:** 2021-03-06

**Authors:** Daniah Al-oufi, Huwidah Mohammad Alkharboush, Nadia Dawood Younis, Ahmed Abu-Zaid

**Affiliations:** 1 Department of Obstetrics and Gynecology, East Jeddah General Hospital, Jeddah, SAU; 2 College of Graduate Health Sciences, The University of Tennessee Health Science Center, Memphis, USA

**Keywords:** vaginal fornix, foreign body, preadolescent child, vaginoscopy

## Abstract

The self-introduction of batteries into the vagina is exceedingly infrequent among preadolescents, with only six cases have been recorded in the English-language PubMed-indexed literature. Herein, we present the case of a five-year-old female child who presented with an 18-month history of recurrent ill-smelling vaginal discharge. Pelvic radiograph displayed a radio-opaque object, most likely representing a disk battery inside her vagina. Vaginoscopy showed a 1.2 cm disk battery that was removed from the right posterior vaginal fornix. Four weeks later, the child was doing well and symptom-free. Although rare, vaginal foreign bodies should be considered in the differential diagnosis in preadolescent girls presenting with chronic and recurrent vaginal discharge. Vaginoscopy is a useful tool diagnostically and therapeutically. A clinical summary of all PubMed-indexed cases of batteries as vaginal FBs in children is provided (n=6).

## Introduction

Vaginal foreign bodies (FBs) are unusual genital complaints. The estimated frequency of vaginal FB incidents is less than 5% in preadolescent females [1]. The vast majority of the cases take place between three and nine years of age [2]. The most frequently documented symptoms comprise vaginal discharge and bleeding [1,3]. A wide array of vaginal FBs has been described, such as nuts, stoppers, pencils, safety pins, and cloths. Nonetheless, clumped toilet paper is the most frequently encountered item in about 80% of all vaginal FB incidents [3]. The self-introduction of batteries into the vagina is exceedingly infrequent, with only six cases of batteries as vaginal FBs have been recorded in the PubMed-indexed literature [2-7]. Herein, we present the case of a five-year-old female child who had a disk battery lodged into the vagina.

## Case presentation

A five-year-old female child presented to our outpatient clinic with an 18-month history of recurrent ill-smelling vaginal discharge. As reported by the mother, there was no apparent dysuria, hematuria, or discomfort. The child was treated with repeated courses of antibiotics for vulvovaginitis; however, there was no substantial therapeutic response. Past medical and surgical histories were unremarkable.

On physical examination, the child was vitally stable and no signs of sexual abuse were suspected. Genital examination revealed erythema and excoriation around the vulva. The yellowish foul-smelling discharge was observed from the vaginal orifice. A digital rectal examination was not carried out. Cultures of the vaginal discharge demonstrated multi-drug resistant Escherichia coli. Subsequently, the child was admitted and started on intravenous cilastatin/imipenem for ten days. Laboratory investigations were notable only for a white blood cell count of 9200 cells/uL (normal range: 4000 to 11,000 cells/uL).

**Figure 1:**
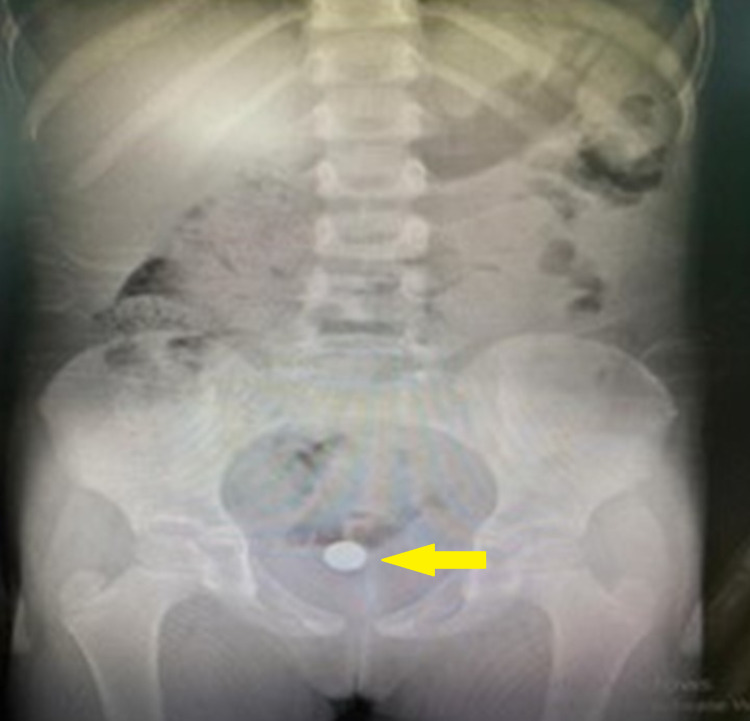
Pelvic radiograph showing a radio-opaque object (yellow arrow), most likely representing a disk battery foreign body at the lower part of the pelvic cavity (vagina).

Pelvic radiograph displayed a radio-opaque object, most likely representing a disk battery FB at the lower part of the pelvic cavity. The exact anatomical position of the FB could not be pinpointed, however, the vagina was the most likely site of involvement (Figure 1).

**Figure 2:**
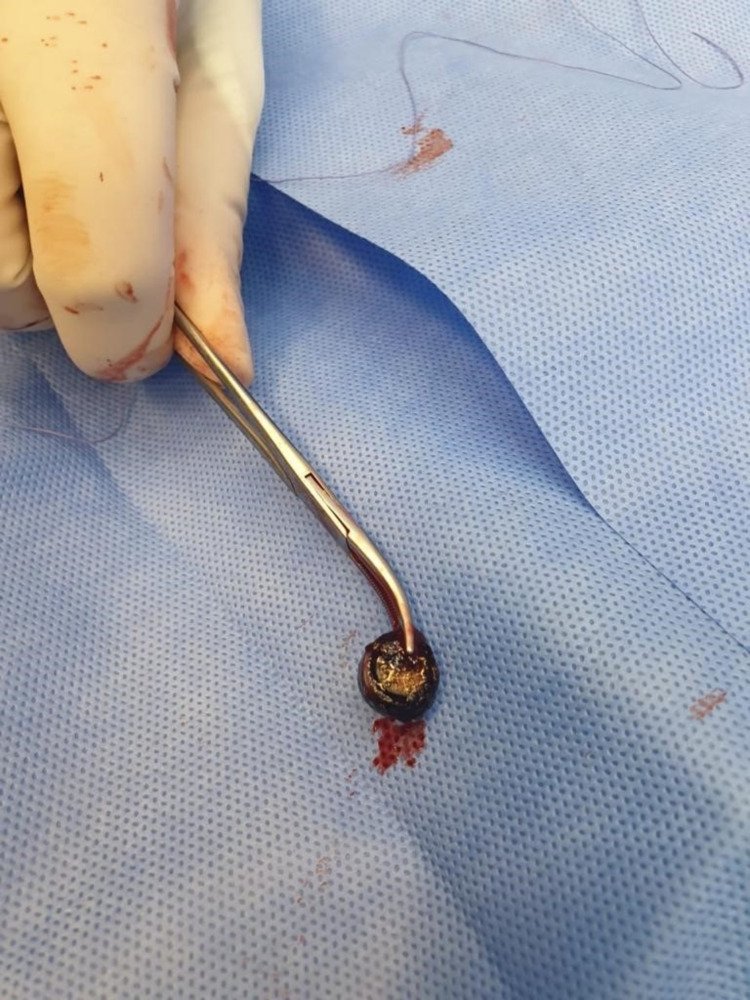
A 1.2 cm disk battery with granulation tissue and no bleeding was removed from the right posterior vaginal fornix during vaginoscopy.

The child underwent vaginoscopy. A 1.2 cm disk battery was removed from the right posterior vaginal fornix without active bleeding. The extracted disk battery had surrounding granulation tissue (Figure 2). The vagina was irrigated with saline several times afterward.

After the completion of the in-patient course of cilastatin/imipenem antibiotics, the child was discharged home in good condition. At four weeks post-discharge, the child was seen in the outpatient clinic and she was doing well and symptom-free. 

## Discussion

Vaginal FBs are infrequent clinical encounters at pediatric outpatient clinics and emergency departments. Bundled pieces of toilet paper are the most frequent FB objects [3]. To the best of our knowledge, only six cases of batteries as vaginal FBs have been recorded in the English-language PubMed-indexed literature (Table 1) [2-7]. Our presented case is the seventh and it enriches the existing scarce literature.

**Table 1:** A summary of all PubMed-indexed cases about batteries as vaginal foreign bodies.

References	First author	Year	Age (year)	Symptoms	Duration	Imaging	Battery type	Battery-induced damage	Management	Follow-up
[4]	Yanoh and Yonemura	2005	12	Fever of unknown origin and lower abdominal pain	72 hours	Radiography, concomitant barium enema and vaginography	Cylindrical	Vaginal ulceration	Battery removal, necrotic debridement, systemic antibiotics, vaginal irrigation	8 weeks, symptom-free and complete healing
[5]	Huppert et al.	2009	13	Abdominal pain, vaginal discharge, and bleeding	42 hours	Cystoscopy, vaginoscopy, sigmoidoscopy	Cylindrical	Necrotic vaginal burn	Battery removal, oral pain killer, topical estrogen, topical cortisone, systemic antibiotics	3 years, symptom-free and complete healing
[6]	Griffin et al.	2015	8	Vaginal bleeding, urinary retention, and dysuria	12 hours	Sigmoidoscopy, radiography	Disk	Vaginal burn	Battery removal, oral pain killer, oral antiemetic, topical estrogen, topical antibiotics, systemic antibiotics	7 days, symptom-free and complete healing
[7]	Semaan et al.	2015	5	Vaginal discharge, pelvic pain	48 hours	Vaginoscopy, cystoscopy, rectoscopy	Disk	Vaginal burn	Battery removal, topical estrogen, systemic antibiotics	1 year, symptom-free, complete healing
[2]	Khan et al.	2016	2.5	history of self-insertion of disk battery	8 hours	Radiography, Vaginoscopy, proctoscopy	Disk	Vaginal burn	Battery removal, vaginal irrigation, topical antibiotic	4 weeks, symptom-free and complete healing
[3]	Nakib et al.	2017	13	Vaginal discharge	6 years	Radiography, ultrasound, computed tomography, vaginoscopy	Disk	Vaginal stenosis	Battery removal, vaginal irrigation, systemic antibiotics	1 day, symptom-free and complete healing
Present	Al-oufi et al.	2021	5	Vaginal discharge	18 months	Radiography	Disk	Vaginal burn	Battery removal, vaginal irrigation, systemic antibiotics	4 weeks, symptom-free and complete healing

In this series of batteries as vaginal FBs (n=7), the median age was eight years (range: 2.5-13 years). This age of presentation is in harmony with the published literature in which the vast majority of vaginal FB cases take place between three and nine years of age [2].

Pelvic/abdominal pain (n=4) and vaginal discharge (n=4) were the most frequently reported symptoms, consistent with the published literature [1,8]. Generally, the presenting clinical manifestations largely depend on the dimension and nature of the introduced vaginal FBs. Additional potential presenting symptomatology of vaginal FBs comprises vaginal bleeding, genital itching, vulvar erythema, and dysuria [1,8]. Five and two cases had disk and cylindrical type batteries inserted into the vagina, respectively. 

It is technically challenging to precisely determine the timeline of vaginal FB occurrence as children may not be able to narrate the history. Nevertheless, few children are smart enough to communicate losing something in their vagina. The duration of presenting complaints can be acute (within days) or chronic (for years). Nakib and colleagues reported the longest duration of six years for a disk-type battery FB lodged into the vagina of a pubescent girl [3]. The short- and long-term residence of lithium/alkaline batteries in the vagina causes battery-induced injuries of the vagina [4]. Such injuries include vaginal ulcerations, burns, fistulas, adhesions, and stenosis [2-7]. Literature review of Table 1 showed that battery-induced physical damages included only burn, ulceration, and stenosis. It appears that the duration of the inserted foreign body affected the type of battery-induced damage; vaginal burn and ulceration occurred acutely whereas stenosis occurred chronically.

Child abuse, urinary tract infection, vaginitis, and vulvitis are possible differential diagnoses in preadolescent children presenting with vaginal discharge [7]. History taking, physical examination, laboratory testing, and imaging modalities are all equally beneficial in ascertaining the most likely diagnosis. The genital examination usually takes place under general anesthesia in consideration of the young age group of children with vaginal FBs. Imaging most often portrays the vaginal FBs. Radio-opaque objects can be easily visualized through radiographs. The selection of imaging modality-namely radiograph, computed tomography, and magnetic resonance imaging-varies and is governed by several factors, one of which is the patient’s age and presenting complaints. In our case, a radiograph was adequate to localize the vaginal FB at the lower part of the pelvic cavity. Vaginoscopy under general anesthesia is a useful diagnostic and therapeutic tool in adolescent children with suspected vaginal FBs [8]. Systemic antibiotics are often recommended and administered [3-7].

Potential complications that can occur if the vaginal FB is missed and not removed in time include vaginal ulcerations, burns, vesicovaginal fistulas, adhesions, and stenosis [2-7]. None of the reviewed cases of batteries as vaginal FBs in children resulted in vesicovaginal fistulas (Table 1). In fact, vesicovaginal fistulas are frequently caused by obstructed labor and following gynecological surgery [9]. While vesicovaginal fistulas occasionally can occur secondary to underlying malignancies, they rarely take place secondary to vaginally inserted FBs [9]. Nonetheless, several cases of vesicovaginal fistulas have been reported in literature among adults (more than 18 years old) [10-13].

## Conclusions

In summary, the self-introduction of batteries as FBs into the vagina is exceedingly infrequent. However, it should be considered in the differential diagnosis in preadolescent girls presenting with chronic and recurrent vaginal discharge. Failure to remove the vaginal FBs early can result in unfavorable acute and chronic complications, such as vaginal ulcerations, burns, vesicovaginal fistulas, adhesions, and stenosis. Vaginoscopy is a useful tool diagnostically and therapeutically in the management of vaginal FBs.
